# Analytical study on metabolic syndrome and psoriasis severity: A cross sectional study

**DOI:** 10.6026/973206300214185

**Published:** 2025-11-15

**Authors:** Amirtha Varshine Navaneeta Krishnan, Achal Ganiga Lingaraj, Amir Kammoun, Shravya Kanishka Mareboina, Sebah Elsa John, Joshua Chacko, Muhammed Nabeel Pullithodika

**Affiliations:** 1 Department of General Medicine, Stanley Medical College Hospital, Chennai, Tamil Nadu, India; 2 Department of General Medicine, Mysore Medical College and Research Institute, Kuvempu, Mysore, Karnataka, India; 3 Department of General Medicine, Sechenov University, Moskva, Russia; 4 Department of General Medicine, General Hospital, Narayanapuram, Alappuzha, Kerala, India; 5 Department of General Medicine, Swansea Bay University Health Board, Baglan, Port Talbot; 6 Department of Dermatology, All India Institute of Medical Sciences, Bibinagar, Telangana, India

**Keywords:** Psoriasis, metabolic syndrome, psoriasis area and severity index (PASI) score, cross-sectional study, obesity, dyslipidemia, disease severity

## Abstract

Psoriasis is a chronic inflammatory skin disease increasingly linked to metabolic syndrome, yet the extent of this association remains unclear. This cross-sectional study examined the relationship between psoriasis severity and metabolic syndrome among 122 adults with chronic plaque psoriasis. Psoriasis Area and Severity Index (PASI) scores were significantly higher in patients with metabolic syndrome, indicating greater disease severity. Central obesity and dyslipidemia were the most prevalent metabolic factors associated with elevated PASI scores. Thus, we show the importance of routine metabolic screening in psoriasis patients to improve disease management and outcomes.

## Background:

Psoriasis is a chronic, immune-mediated, inflammatory disease of the skin, affecting about 2-3% of the world's population [1]. In addition to its cutaneous effects, psoriasis is increasingly being recognized as a systemic disease associated with comorbidities, such as insulin resistance, cardiovascular disease and features of the metabolic syndrome (MetS) [2]. MetS is defined by the presence of one or more of the following: central obesity, hypertension, hyperglycemia, low HDL cholesterol and hypertriglyceridemia [3]. Patients with moderate to severe psoriasis often exhibit these inflammatory metabolic pathways [4]. The proposed pathophysiological mechanism between psoriasis and MetS is believed to be bidirectional, via overlapping inflammatory pathways, especially with cytokines such as tumor necrosis factor-alpha (TNF-α), interleukin-6 (IL-6) and interleukin-17 (IL-17) [5]. This relationship may lead to increased systemic inflammation, exacerbation of skin lesions and possibly not responding as well to treatment [6]. However, while there are associations, clinical studies have yielded mixed results regarding psoriasis severity and the MetS status [7]. The increasing prevalence of both diseases and their influence on public health, imposes a need to understand the association of metabolic dysfunction on psoriasis severity [8]. Therefore, it is of interest to report the prevalence of metabolic syndrome among patients with psoriasis and to evaluate its association with disease severity as measured by Psoriasis Area and Severity Index (PASI) scores.

## Materials and Methods:

A year-long analytical cross-sectional study was conducted at a tertiary care outpatient dermatology department. In all, 122 individuals between the ages of 18 and 65 who had been diagnosed with persistent plaque psoriasis were enrolled in succession. Exclusion criteria included patients with psoriatic arthritis, other forms of psoriasis (guttate, pustular, erythrodermic), on-going systemic infections, or those on systemic steroids or biologics within the past 8 weeks. Each participant underwent a thorough clinical examination, including measurement of weight, height, waist circumference and blood pressure. Body Mass Index (BMI) was calculated and laboratory investigations were performed, including fasting blood glucose, triglycerides and HDL cholesterol. Metabolic syndrome was diagnosed according to the modified NCEP ATP III criteria (Asian waist circumference cut-offs). Psoriasis severity was assessed using the Psoriasis Area and Severity Index (PASI) and patients were categorized into mild (PASI <10), moderate (10-20) and severe (>20) disease. The sample was stratified into two groups: those with MetS and those without. Data were analyzed using SPSS version 26.0. Quantitative variables were expressed as means and standard deviations and comparisons between groups were made using t-tests and chi-square tests. A p-value of <0.05 was considered statistically significant.

## Results:

A total of 122 patients with psoriasis were studied, where 58 (47.5%) patients had metabolic syndrome (MetS). Patients with MetS were significantly older with higher BMI and waist circumference than those without MetS. The severity of psoriasis assessed by PASI was significantly higher for patients with MetS, where severe psoriasis was also observed higher in these patients. Cases of dyslipidemia, elevated triglycerides and impaired fasting glucose were also higher in cases of severe psoriasis, indicating a possible link to MetS. A dose-response relationship was established between the total number of MetS components and PASI scores in an apparent stepwise increase, while hypertension also emerged as strongly associated with increased psoriasis severity. The duration of disease was notable, with the highest incidence of MetS occurring in patients with 10 or more years' duration of psoriasis. Increased BMI strongly associated with increased PASI scores would further support obesity as a contributing factor. Males had a slightly higher prevalence than females for MetS and more severe psoriasis. Significant systemic symptoms of fatigue and joint pain were also reported more frequently in patients with MetS. Patients with metabolic syndrome had significantly higher PASI scores, greater disease severity and more frequent central obesity and dyslipidemia compared to those without metabolic syndrome. A clear association was observed between the number of metabolic components and psoriasis severity. Table 1 (see PDF) shows that patients with metabolic syndrome were older, had higher BMI and greater waist circumference compared to those without MetS. Table 2 (see PDF) indicates that patients with metabolic syndrome had significantly higher PASI scores, reflecting more severe psoriasis. Table 3 (see PDF) demonstrates that severe psoriasis was considerably more frequent in the metabolic syndrome group. Table 4 (see PDF) highlights that dyslipidemia and elevated fasting glucose were significantly more common in patients with severe psoriasis. Table 5 (see PDF) shows a stepwise increase in PASI scores with rising numbers of metabolic syndrome components. Table 6 (see PDF) reveals that hypertension prevalence increased with psoriasis severity, being most common in severe cases. Table 7 (see PDF) indicates that metabolic syndrome was more frequent in patients with psoriasis of more than 10 years' duration. Table 8 (see PDF) shows that higher BMI categories were strongly associated with greater PASI scores. Table 9 (see PDF) demonstrates that male patients had a slightly higher prevalence of metabolic syndrome and higher PASI severity scores than females. Table 10 (see PDF) shows that systemic symptoms such as fatigue and joint discomfort were more common among patients with metabolic syndrome.

## Discussion:

This analytical cross-sectional study establishes a significant association between metabolic syndrome and increased severity of chronic plaque psoriasis. Patients with metabolic syndrome exhibited higher PASI scores, more frequent systemic symptoms and a greater prevalence of risk factors such as obesity, hypertension and dyslipidemia [9]. These findings are consistent with prior research suggesting that the chronic low-grade inflammation underlying metabolic syndrome may amplify psoriatic inflammation through shared cytokine pathways, including TNF-α, IL-6 and IL-17 [10]. One of the most striking observations in our study was the strong correlation between the number of metabolic syndrome components and psoriasis severity [11]. As the number of metabolic derangements increased, PASI scores rose correspondingly. This reinforces the hypothesis of a dose-dependent inflammatory burden that worsens skin involvement. Furthermore, high BMI and waist circumference were significantly associated with more severe disease, emphasizing the need for weight management as a part of integrated psoriasis care [12]. Hypertension and elevated fasting glucose, both common among psoriatic patients in our study, also corresponded with higher CRP levels and systemic symptoms, suggesting a systemic inflammatory spillover beyond skin lesions [13]. Interestingly, longer disease duration was linked to higher metabolic syndrome prevalence, raising the question of whether persistent inflammation over time contributes to the development of metabolic dysregulation-or vice versa [14]. Lifestyle factors such as tobacco and alcohol use were significantly more common among patients with both severe psoriasis and metabolic syndrome. These modifiable risk factors may exacerbate both conditions and should be prioritized in patient counselling [15]. The higher prevalence of systemic symptoms (e.g., fatigue and joint discomfort) in patients with metabolic syndrome points toward an emerging phenotype of "systemic psoriasis," where dermatologic manifestations are only one aspect of a broader inflammatory profile [16]. While this study provides valuable insights, it is limited by its cross-sectional design, which precludes causal inference. Additionally, it did not assess longitudinal disease progression or therapeutic response. However, the strength of the association between metabolic syndrome and psoriasis severity suggests that routine screening for metabolic abnormalities should be considered in all patients with moderate to severe psoriasis.

## Conclusion:

Metabolic syndrome is significantly linked to greater psoriasis severity through its systemic inflammatory effects. Key components such as obesity, dyslipidemia and hyperglycemia further exacerbate psoriatic inflammation. An integrated approach addressing both dermatologic and metabolic factors is essential for optimal patient management.
